# Implementation of a National Prenatal Exome Sequencing Service in England: Cost‐Effectiveness Analysis

**DOI:** 10.1111/1471-0528.18020

**Published:** 2024-11-21

**Authors:** Emma J. Smith, Melissa Hill, Michelle Peter, Wing Han Wu, Corinne Mallinson, Steven Hardy, Lyn S. Chitty, Stephen Morris

**Affiliations:** ^1^ NHS North Thames Genomic Laboratory Hub Great Ormond Street Hospital for Children NHS Foundation Trust London UK; ^2^ Genetics and Genomic Medicine UCL Great Ormond Street Institute of Child Health London UK; ^3^ National Disease Registration Service National Health Service England London UK; ^4^ Department of Public Health and Primary Care University of Cambridge Cambridge UK

**Keywords:** budget impact, cost‐effectiveness, economic evaluation, exome sequencing, genomics, prenatal

## Abstract

**Objective:**

Prenatal exome sequencing (pES) for diagnosing fetal structural anomalies commenced in the English National Health Service (NHS) in 2020. We evaluated cost‐effectiveness to the healthcare system, and costs to families, of pES in addition to standard testing, compared to standard testing alone.

**Design:**

A cost‐effectiveness analysis combining costs, outcomes, parent and professional interview and professional survey data.

**Setting:**

The English NHS Genomic Medicine Service.

**Sample:**

413 families with fetal anomalies with a suspected genetic cause referred for pES from 01 October 2021 to 30 June 2022.

**Methods:**

We costed the incremental resource required to deliver the pES clinical pathway. We calculated the diagnostic yield (proportion of cases with pathogenic variants). We divided the total incremental cost by the number of cases with a diagnosis to calculate cost‐effectiveness. We estimated the annual NHS budget requirement based on case numbers. We determined parental costs from interviews.

**Main Outcome Measures:**

Incremental costs of pES to the NHS and families, incremental cost per additional diagnosis and NHS budget impact.

**Results:**

Of 413 referred cases, 241 were tested, at a cost of £2331 (95% credibility interval £1894–£2856) per referred case or £3592 (£2959–£4250) per case that proceeded with testing. The incremental cost per diagnosis (yield 35.3%) was £11 326 (£8582–£15 361). Based on referrals data 01 October 2022 to 30 September 2023, pES costs the NHS £1.8 m annually. Family costs could not be separated from other pregnancy‐related appointments but were not considered burdensome; most appointments were concurrent or remote.

**Conclusion:**

pES costs the English NHS £11 326 for each additional diagnosis. Incremental costs to families are negligible.

## 1 Introduction

**FIGURE 1:**
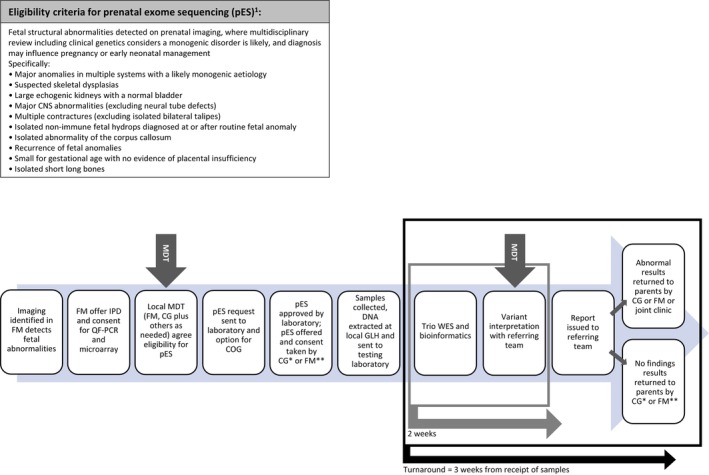
Overview of the pES pathway. Local pathways can vary in which staff groups are involved in taking consent and return of results (from Peter et al.) CG, Clinical genetics; COG, Clinical oversight group; FM, Fetal medicine; GLH, Genomic laboratory hub; IPD, Invasive prenatal diagnosis; MDT, Multi‐disciplinary team; QF‐PCR, Quantitative fluorescent‐polymerase chain reaction; WES, Whole exome sequencing; *, may include genetic counsellors; **, may include midwives.

Prenatal exome sequencing (pES) was introduced in the English National Health Service (NHS) Genomic Medicine Service (GMS) in October 2020 [1, 2] based on evidence of its potential benefits [3, 4, 5]. pES is available when there are no informative findings from standard testing (rapid aneuploidy exclusion and chromosomal microarray [CMA]) to families that have anomalies with a suspected genetic cause identified on fetal ultrasound (Figure 1), where results have the potential to influence pregnancy management [1]. This includes refining the diagnosis to allow better prediction of prognosis to facilitate parental decisions on termination, and other management decisions such as place of delivery, neonatal follow‐up care required. Testing in the NHS GMS is delivered through seven regional Genomic Laboratory Hubs (GLHs) [6]. All seven conduct the standard prenatal testing for families in their region, with two GLHs performing pES for the whole country.

pES is ordered following negative aneuploidy testing (via QF‐PCR) and in parallel with, or subsequent to, CMA. pES only proceeds to reporting if CMA is negative. It is requested by clinical genetics professionals following multidisciplinary team (MDT) discussion involving fetal medicine professionals. The request is sent to one of the two testing GLHs (depending on geographic region) and reviewed for eligibility. Referrals are rejected if the cause is not thought to be genetic, fetal demise or termination of pregnancy is imminent, another more appropriate genetic test is available, or parents decline invasive testing. If accepted by the laboratory, the parents are consented by either clinical genetics or foetal medicine professionals. Parental blood samples are taken and DNA extracted for trio sequencing (foetal DNA already obtained for QF‐PCR and CMA testing is used). Results are returned by clinical genetics or foetal medicine, or jointly (Figure 1; [7]). This is the first example of a national pES service globally. Costs and outcomes are yet to be evaluated outside of a research setting [8, 9]. The aim of this Optimising EXome PREnatal Sequencing Services (EXPRESS) [10] study was to identify the incremental costs of delivering pES to the NHS and to families, compared to standard testing only, and to synthesise this with key outcomes data to produce a cost‐effectiveness analysis.

## 2 Methods

### 2.1 Overview

We derived costs of pES from the clinical pathway (Figure 1), identifying and quantifying the incremental resource to deliver pES in addition to standard testing, compared to standard testing alone. As standard testing was performed in both the intervention and comparator scenarios the additional costs and outcomes specifically associated with pES represent the total incremental costs and outcomes. The perspective was the English NHS and costs were calculated in 2021–2022 UK pounds. The time horizon was the duration of pregnancy. We also collected outcomes data and divided incremental cost by additional diagnoses from pES to ascertain cost‐effectiveness. We sought patient reported costs through interviews and summarise the findings.

### 2.2 Sample

The sample was all cases (*n* = 413) referred for pES to the two testing GLHs during the study period (01 October 2021 to 30 June 2022). Population characteristics are provided at Table S1.

### 2.3 Costs

We identified and costed the key stages of the pathway: case identification and referral to the testing GLH, discussion and consent, sample collection, transportation and DNA extraction (parental samples), pES, return of results and administration.

We collected data on resource use through surveys with clinical genetics and foetal medicine professionals (methodology reported elsewhere [7]). Professionals were asked to identify incremental resources to deliver pES including extra time spent discussing and taking consent, number of additional appointments and time spent on associated administration. They were also asked to identify which staff groups were usually responsible for undertaking each process. For our base case, we aggregated the survey data from five (out of 17) sites identified as using the most common service delivery model delineated by Walton et al. [11] The core staff were a fetal medicine consultant, clinical geneticist, clinical scientist and genetic counsellor.

We calculated the mean incremental staff time for each stage and applied unit staff costs using the NHS Agenda for Change pay scale and (for consultant grades) the Personal Social Services Research Unit Costs Database of Health and Social Care Professionals [12, 13], with on‐costs (national insurance and NHS pension) at prevailing rates and overheads at 25%. We calculated a weighted average cost by applying the percentage of each staff group delivering each part of the pathway, from the survey data. For administration, which was estimated by respondents for the pathway as a whole, we applied the same weighting as for discussion and consent. The laboratory cost of pES was the mean of costings prepared by the two testing GLHs for the purpose of establishing reimbursement. We supplemented costs with published data where appropriate. We calculated the incremental cost to the NHS of pES (compared to no pES) by applying the cost per case for each pathway stage to the number of cases proceeding through that stage. We calculated the mean incremental cost per pES referral by dividing the total cost by the total number of cases referred.

Our semi‐structured interviews with parents [14] included a section on the financial costs of pES to themselves and their family. Questions covered appointment format (i.e., in person or video/telephone consultation), travel, childcare arrangements and time off work. Responses were analysed as described elsewhere [14].

### 2.4 Outcomes

Outcomes data were collected from the two testing GLHs and referring clinical genetics services. These were linked to data collected by the National Congenital Anomaly and Rare Disease Registration Service and the national Maternity Services Data Set (linkage methodology described elsewhere [15]). The key outcome was identified as diagnostic yield, defined as at least one pathogenic (or likely pathogenic) variant reported as a result of pES. This was used to calculate the incremental cost per additional diagnosis.

Additional analysis was performed using data from the two testing GLHs to consider whether the result had changed clinical management or pregnancy continuation decision and the frequency of incidental findings. We did not include pregnancy outcomes in our analysis because it was not possible to obtain linked pregnancy outcome data for our entire sample.

### 2.5 Scenario and Sensitivity Analysis

We conducted probabilistic sensitivity analysis, running 1000 simulations with gamma distributions for costs and beta distributions for probabilities (of a case proceeding and of receiving a diagnosis). We drew a cost‐effective acceptability curve showing the probability pES is cost effective, versus no pES, at different willingness‐to‐pay levels for an additional diagnosis. We calculated 95% credibility intervals using the 2.5th and 97.5th percentiles of the simulations.

To assess the impact of using different service delivery models, we identified two most common sub‐types from the seven models described [11]; those with only consultant grade core staff (‘scenario 1’) and those that included a midwife within core staff (‘scenario 2’). We aggregated the survey responses relating to each of these model types and calculated the mean resource use, applying costs as described above. We also calculated the mean diagnostic yield for each scenario using outcome data by referral centre and applied this to calculate the cost per diagnosis. Two further scenarios (three and four) examined the impact of the highest and lowest diagnostic yield recorded across all seven GLH regions.

### 2.6 Budget Impact Analysis

We obtained pES case numbers from the testing GLHs for a recent 12‐month period (01 October 2022 to 30 September 2023) representative of current demand as our study period was soon after roll‐out, when referrals may not have reached steady state. We multiplied proceeded and non‐proceeded case numbers by the mean cost per proceeded/non‐proceeded case respectively and aggregated these to estimate the annual incremental cost to the NHS of the pES service.

### 2.7 Threshold Analysis Comparing pES and Prenatal Genome Sequencing

Prenatal genome sequencing (pGS) may have additional benefits compared to pES including reduced turnaround time, more even sequencing coverage, ability to perform copy number variant (CNV) analysis and ability to detect disease‐causing variants in non‐coding regions [16, 17, 18]. We calculated the maximum cost at which pGS would be no more expensive overall than pES, taking account of the saving from cessation of CMA testing on the assumption CNV analysis would replace this. The mean cost of a prenatal CMA was calculated using costings obtained from four GLHs (out of seven) that responded to our information request.

### 2.8 Patient and Public Involvement

Patient and public involvement (PPI) in the design of this analysis was via the participation of a PPI Advisory Group in all aspects of EXPRESS, including the development of the recruitment strategy and study materials for parent interviews from which we have analysed data [10].

## 3 Results

During the 9‐month study period, 413 cases were referred to the testing GLHs for pES. Of these, 241 cases proceeded with pES and had a result returned, of which 85 received a diagnosis. Reasons for not proceeding included failure to meet eligibility criteria (*n* = 73) or alternative testing suggested (*n* = 10), pregnancy ended due to fetal demise or termination (*n* = 52) and parents declining invasive testing or pES (*n* = 16). For nine cases testing was started but subsequently transferred to the non‐urgent pathway following fetal demise or termination (post‐sequencing costs and outcomes were excluded from our data to ensure comparability).

In our base case, the incremental cost to the NHS to deliver pES for all 413 cases was £962 727 (95% credibility interval [CI] £775 454–£1 204 027) (Table 1; Table S2). Of the total cost, £865 699 (90%) related to proceeded cases and £97 028 (10%) to non‐proceeded cases. The pES test was £2931 (range £2845–£3009) per case and accounted for the majority of overall cost (76%). The average additional clinical time spent in existing appointments providing counselling about pES was 32 min per case at a total cost of £9935 (1% of overall cost). There were on average an additional 1.9 clinical genetics appointments needed at a cost of £36 498 (4% of total cost), which for simplicity we assumed related to return of results (pre‐test discussion and counselling generally took place in existing fetal medicine appointments). Non‐proceeded case costs included discussion and selection (via MDT) and eligibility review by testing GLHs (£59 881). The mean cost per referred case was £2331 (95% CI £1894–£2856), £3592 (£2959–£4250) for a proceeded case, £564 (£154–£774) for a non‐proceeded case. The diagnostic yield was 35.3% (*n* = 85 cases); therefore, the incremental cost per additional diagnosis was £11 326 (£8582–£15 361) (Table 2).

**TABLE 1:** Cost of delivering prenatal exome sequencing (pES).

Process	Cost per case (£) (95% CI)	No. of cases	Total cost (£)
Case identification and referral to GLH (MDM)	338	413	139 543
Eligibility review by GLH (rejected/non‐proceeded)^a^	11	163	1766
Discussion and consent^b^	40	250	9935
Sample collection, transport and DNA extraction^b^	76	250	18 887
Prenatal exome sequencing (pES)^b^	2931	250	732 733
Return of results	151	241	36 498
Administration (throughout)	57	413	23 366
Total cost (95% credibility interval)			962 727
			(775 454–1 204 027)
**Mean cost**			
Referred pES case	2331 (1894–2856)	413	
Proceeded pES case	3592 (2959–4250)	241	
Non‐proceeded pES case	564 (154–774)	172	

^a^ For accepted cases this cost is included in pES.

^b^ 241 proceeded pES cases, nine cases started then subsequently transferred to non‐urgent pathway.

**TABLE 2:** Cost per outcome and scenario analysis.

	Total cost (£)	Cases diagnosed (out of *n* = 241 proceeded cases)	Cost per diagnosis (£)	Diagnosed and changed management	Cost per diagnosed case with changed management (£)	Proceeded and changed management	Cost per proceeded case with changed management (£)
Base case	962 727	85	11 326	70	13 753	152	6334
Scenario 1—consultant core staff only	963 625	86	11 205				
Scenario 2—core staff includes midwife	955 289	84	11 372				
Scenario 3—high diagnostic yield	962 727	110	8752				
Scenario 4—low diagnostic yield	962 727	69	13 953				

*Note:* Changed management outcome rates not re‐calculated for scenarios due to potential bias from low case numbers and response rates of some GLHs/referral units.

**FIGURE 2:**
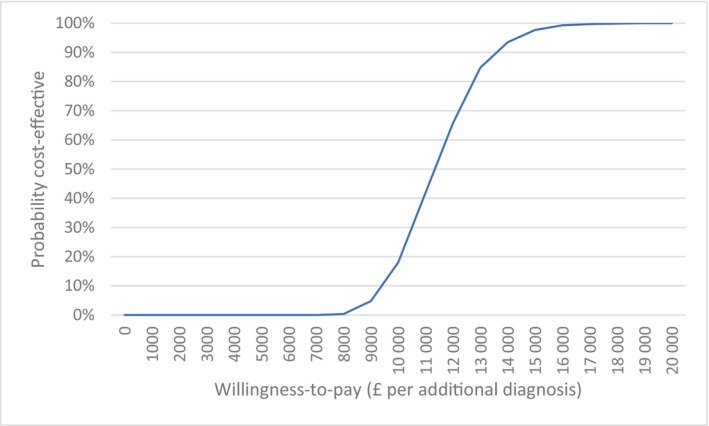
Cost‐effective acceptability curve showing probability pES is cost effective for different willingness‐to‐pay levels for an extra diagnosis.

The cost‐effectiveness acceptibility curve (Figure 2) shows that for willingness‐to‐pay thresholds of £8000, £12 000 and £14 000 per additonal diagnosis, 0%, 66% and 94%, respectively, of the 1000 simulations were cost‐effective.

### 3.1 Scenario Analysis

Given that pES made the largest contribution to additional costs, the alternative staffing scenarios had a negligible impact on diagnostic yield and costs (Table 2). For our first scenario—delivery models that did not include either a genomic counsellor or a midwife within the core team—the total cost was estimated to be £963 625, 0.1% higher than the base case. The unit staff costs were higher due to the greater proportion of consultant level staff used, but less additional time was required for existing appointments (29 compared to 32 min) and there were slightly fewer additional appointments (1.8 vs. 1.9). The diagnostic yield was 35.7% (vs. 35.3% in the base case) resulting in a cost per diagnosis of £11 205. For scenario two—delivery models involving a midwife in the core team—the total cost was £955 289, 0.8% lower than the base case. Additional time in existing appointments was slightly higher at 34 min, while the number of additional clinical genetics appointments to return results was lower at 1.4, possibly due to a greater proportion of results being returned and support given by foetal medicine. Diagnostic yield was 34.9% resulting in cost per diagnosis of £11 372.

The result was more sensitive to diagnostic yield variations; taking the base case cost and applying the highest (45.5%) and lowest (28.6%) diagnostic yields found across referring GLHs, the incremental cost per additional diagnosis was £8752 and £13 953, respectively.

### 3.2 Alternative Outcomes

We collected outcomes data from clinicians on the impact of pES on clinical management. ‘Changed management’ was defined as: influenced decision to continue or end pregnancy, influenced medical management during pregnancy, prenatally or neonatally, more than one of these factors or other (with comments). Responses were obtained for 42% of proceeded cases. Of the cases that received a diagnosis 82% reported a change in management. Extrapolating for total diagnosed cases (*n* = 85) we estimated that *n* = 70 cases received a clinically useful diagnosis. Taking the base case cost, the cost per outcome was therefore £13 753 (Table 2). However, responses also showed that for 51% of pES cases without a diagnosis (i.e. no findings or a variant of uncertain significance), there was a change in management. Across all proceeded cases management was therefore estimated to have changed for 63%; extrapolating this to our total proceeded cases sample (*n* = 241), we estimated that 152 cases had a change in management. By this measure, the cost per outcome reduced to £6334.

Incidental findings were reported in 2.9% (*n* = 7) of proceeded cases including a variety of rare conditions warranting clinical review in the parent(s) or impacting future reproductive risks. Incremental costs and benefits would be expected to accrue from downstream standard clinical follow‐up, but we have not included these in our analysis.

### 3.3 Annual Incremental NHS Cost

In the 12 months to 30 September 2023, the testing GLHs received 760 referrals, 442 of which proceeded to pES testing. Applying the average cost per proceeded (*n* = 442) and non‐proceeded (*n* = 318) case, the latter including costs for *n* = 17 assumed sequenced then transferred to the non‐urgent pathway (based on 9/172 [5%] cases in our study sample) the total annual incremental cost to the NHS of delivering a pES service was estimated to be £1 768 193 (Table S3).

### 3.4 Prenatal Genome Sequencing

The mean cost of a prenatal CMA was £352. On the assumption this would be replaced by CNV analysis in every pGS case, the cost of pGS could be up to £3283 for this testing approach to be no more expensive overall than pES.

It is possible there would be a higher number of cases that proceed with pGS compared to pES in the absence of CMA testing; in our sample, one case was declined by the testing GLH and three were accepted but subsequently discontinued due to a pathogenic finding from a CMA (or other) test. Under a pGS approach, it is possible that all four cases (accounting for 1% of referrals) would have proceeded. This would have resulted in additional costs of £12 791 (1.3% of the total base case cost).

### 3.5 Patient Costs

Responses from semi‐structured interviews with parents (42 women and 6 male partners) about the financial cost of pES were analysed. It was difficult to identify the costs associated with pES specifically as, due to having anomalies identified in pregnancy, this group was already frequently attending hospital for scans and monitoring and respondents did not necessarily distinguish between these and pES related appointments. Consequently, it was not possible to quantify the cost to families as we could not confidently ascribe disclosed costs to pES.

When talking about travel expenses, childcare and time off work related to pregnancy appointments in general, there was a lot of variability in costs that were disclosed based on parents' proximity to the hospital and job flexibility. The pES pre‐test discussion mostly took place in person while already attending hospital for a scan, with bloods taken at the same time. Two people described a remote pre‐test discussion (via video/telephone call) followed by attending the local hospital for phlebotomy. Most pES results were returned remotely: phone call (*n* = 22), in person (*n* = 8), virtual (*n* = 4) and letter (*n* = 1) (*n* = 4 unknown, *n* = 3 not applicable). Several parents noted that they did not think they had any additional expenses specific to pES.

## 4 Discussion

### 4.1 Main Findings

We estimated the incremental cost to the NHS to deliver pES during the period 1 October 2021 to 30 June 2022 to be £962 727 for a total of 413 referred cases, 241 of which proceeded with pES and 85 received a diagnosis. The mean cost per case referred was £2331 and the incremental cost per additional diagnosis was £11 326. We also considered an alternative outcome measure—whether or not pES changed the clinical management and/or pregnancy continuation decision—and found that it did in 63% of proceeded cases (82% of cases with a diagnosis and 51% without). The cost per ‘changed management’ outcome was £6334.

We analysed the impact of different pES service delivery models based on core staffing. There were some cost variances due to the relative salaries of staff involved and incremental time/appointments to deliver pES; however, as the majority (76%) of the cost was attributable to the pES test itself (which does not depend on the delivery model), these differences had a negligible impact on overall cost. There was also a negligible difference in diagnostic yield and consequently cost per diagnosis between the models. There was a more significant impact when the diagnostic yield was varied (which it did across GLHs, from 28.6% to 45.5%) with the cost per diagnosis ranging from £8752 to £13 953.

Applying recent, steady state pES referral numbers, the total annual incremental cost to the NHS of delivering a pES service is estimated to be £1.8 m. If pES were replaced with pGS (similarly to rapid paediatric sequencing [1, 19]), the cost of the test would need to be no more than £3283 per case to require no additional NHS budget, assuming clinical pathways would otherwise remain unchanged and CMA testing would not be required alongside.

We were unable to quantify the average cost of pES to families as interview respondents did not always differentiate expenses from existing pregnancy‐related appointments. However, responses indicated that counselling and phlebotomy did not usually require an additional hospital visit, and results were mostly delivered remotely, thus additional costs were minimal or nil for most families.

### 4.2 Strengths and Limitations

This is the first economic analysis of pES in a live clinical service [8, 9]. Costs were sourced directly from health professionals from across England and from the laboratories conducting pES, providing robust estimates of resource use and taking account of local variation in service delivery models.

There were some limitations relating to the laboratory and health professional survey data collected. Our database only captured pES cases that were referred to GLHs, thereby excluding costs for cases assessed by fetal medicine but not subsequently referred. Only clinical genetics professionals were asked to estimate the number of extra appointments for pES, potentially leading to an underestimate of costs where results were returned by fetal medicine (though this may have been done in existing appointments). There was also a low (42%) response rate to the request for clinical genetics professionals to provide pregnancy outcome data, including ‘changed management’ data, and while constraints on clinician time is a factor, clincians that felt pES impacted upon patient care or decision making may have been more likely to respond, potentially biasing this outcome measure.

We did not take into account the costs of pregnancy outcomes as it was not possible to identify a comparator cohort from the national dataset due to insufficient granularity in clinical phenotype descriptors. Our analysis also excludes downstream costs and benefits from incidental findings (identified in 2.9% of cases) and, for diagnosed cases with a live birth outcome, the savings and health benefits that may arise from avoiding a lengthy diagnostic odyssey.

### 4.3 Interpretation

We have found implementation of pES in a live clinical service to be more cost effective than previously shown in a research setting, where the cost per additional diagnosis for pES in addition to CMA was found to be £25 581 [8]. The difference is mainly due to the lower diagnostic yield in the previous study (12.1%) in which broader testing eligibility criteria were applied. Costs additionally included pregnancy outcomes, however, the overall incremental cost was lower, likely driven by the lower pES cost (£2100 vs. £2931).

Compared to other, previously commissioned genetic diagnostic technologies pES is more expensive. CMA testing was found to have a mean incremental cost of £113 with a cost of £4703 per additional pathogenic result compared to karyotyping (the previous standard test) at 2012–13 prices [20]. Based on annual case numbers [21], CMA also impacted less on the NHS budget at approximately £416 k per annum (2021–22 prices) compared to £1.8 m for pES.

Our findings do not indicate a preferred service delivery model among those in use in the NHS GMS; alignment of models would not significantly impact upon costs. However, there is variability in diagnostic yield across GLHs and consistent application of eligibility criteria and access to expert prenatal genetic and fetal medicine services may help reduce variation. Policy makers should consider that more or less stringent application of the eligibility criteria would impact upon the mean cost per diagnosis.

The ‘changed management’ outcome we captured is, arguably, a more encompassing measure of utility than diagnostic yield alone. This is supported by our parent interviews that indicated that pES informs decision making irrespective of result; however, consideration of potential misunderstanding a negative result is needed, as cautioned by health professionals [14]. pES can also inform future reproductive choices and, for live births with a pES diagnosis, there are likey to be downstream savings and health benefits resulting from a foregone, potentially lengthy diagnostic odyssey. This has been demonstrated for children and adults following a genome sequencing diagnosis [22]; the benefits from a prenatal diagnosis could be even greater. Policy makers may wish to consider that benefit may be derived from pES beyond simply the diagnosis.

Qualitative responses from our parent interviews suggested that the incremental costs of pES to families are negligible; they were often at hospital anyway and additional appointments specific to pES were usually remote, minimising travel costs and time away from work or caring responsibilities. Remote consultations for other genetic testing services have been found to be an acceptable modality and more convenient to patients [23, 24].

## 5 Conclusion

The incremental cost of the pES service to the NHS is estimated to be £1.8 m annually, with a mean cost of £11 326 per diagnosis. There is no preferred core‐staffing delivery model among those in use within the NHS GMS on the basis of cost. Additional costs to families are difficult to differentiate from those already incurred for other pregnancy‐related appointments and were therefore found to be negligible. Further research into the potential savings and health benefits arising from the foregone diagnostic odyssey resulting from a prenatal genomic diagnosis may be informative to policy makers.

## Author Contributions

E.J.S.: data curation, formal analysis, methodology and writing – original draft. M.H.: conceptualisation, data curation, methodology, funding acquisition, project administration, supervision and writing – review and editing. M.P.: data curation, formal analysis and writing – review and editing. W.H.W.: data curation, project administration and writing – review and editing. S.H.: data curation, methodology and writing – review and editing. C.M.: data curation, methodology and writing – review and editing. L.S.C.: conceptualisation, funding acquisition, data curation, formal analysis, methodology, supervision and writing – review and editing. S.M.: conceptualisation, funding acquisition, data curation, formal analysis, methodology, supervision and writing – review and editing.

## Ethics Statement

Ethical approval to conduct the interviews with parents was given by the Health Research Authority (HRA) and the East of Scotland Research Ethics Service REC 1 (21/ES/0073). The HRA classified the interviews and surveys with professionals as Service Evaluation and research ethics committee approval was not required. The service evaluation was registered with Research and Development at Great Ormond Street Hospital for Children NHS Foundation Trust. Clinical audits for data collection of pregnancy outcomes were registered for North Thames GLH (GOSH: Reference Number: 3082) and Central and South GLH (Clinical Audit Registration and Management System (CARMS): Birmingham Women's Hospital: CARMS‐31001).

## Conflicts of Interest

The authors declare no conflicts of interest.

## Supporting information


Tables S1–S3.

## Data Availability

The data that support the findings of this study are available from the corresponding author upon reasonable request.
