# Supplementary material for: Implementation of a National Prenatal Exome Sequencing Service in England: Cost‐Effectiveness Analysis
Source: BJOG. 2024 Nov 21;132(4):483–91. doi: 10.1111/1471-0528.18020 (PMC11794059; doi:10.1111/1471-0528.18020)
Supplement: Supplementary file 1 — Tables S1–S3. [file BJO-132-483-s001.docx]

**Table S1 – Population characteristics for all cases in our sample (n = 413) that were referred for prenatal exome sequencing (pES) and either proceeded with testing or did not**

|  | **Proceeded** | | |  | | | **Non-proceeded** | | | | | | **Total** | | |  | | |  |  |  |
| --- | --- | --- | --- | --- | --- | --- | --- | --- | --- | --- | --- | --- | --- | --- | --- | --- | --- | --- | --- | --- | --- |
| **Maternal age** | n | | | % | | | n | | % | | | | n | | | % | | |  | |  |
| < 20 | 7 | | | 2.9% | | | 5 | | 3.2% | | | | 12 | | | 3.0% | | |  |  |  |
| 20 < 25 | 28 | | | 11.6% | | | 11 | | 7.1% | | | | 39 | | | 9.9% | | |  |  |  |
| 25 < 30 | 61 | | | 25.3% | | | 37 | | 24.0% | | | | 98 | | | 24.8% | | |  |  |  |
| 30 < 35 | 85 | | | 35.3% | | | 46 | | 29.9% | | | | 131 | | | 33.2% | | |  |  |  |
| 35 < 40 | 44 | | | 18.3% | | | 40 | | 26.0% | | | | 84 | | | 21.3% | | |  |  |  |
| >40 | 16 | | | 6.6% | | | 15 | | 9.7% | | | | 31 | | | 7.8% | | |  |  |  |
| *missing* | *0* | | |  | | | *18* | |  | | | | 18 | | |  | | |  |  |  |
| Total | 241 | | |  | | | 172 | |  | | | | 413 | | |  | | |  |  |  |
| **Maternal ethnicity** |  | | | |  | | |  | | |  | | |  | | |  | | |  |  |
| White | 153 | | | | 74.6% | | | 28 | | | 82.4% | | | 181 | | | 75.7% | | |  |  |
| Non-white | 52 | | | | 25.4% | | | 6 | | | 17.6% | | | 58 | | | 24.3% | | |  |  |
| *missing* | *36* | | | |  | | | *138* | | |  | | | *174* | | |  | | |  |  |
| Total | 241 | | | |  | | | 172 | | |  | | | 413 | | |  | | |  |  |
| **Index of multiple deprivation** | | |  | | |  | | | |  | |  | | |  | | |  |  |  |  |
| 1 (most deprived) | | 26 | 11.5% | | | 18 | | | | 13.8% | | 44 | | | 12.3% | | |  |  |  |  |
| 2 | | 28 | 12.3% | | | 16 | | | | 12.3% | | 44 | | | 12.3% | | |  |  |  |  |
| 3 | | 23 | 10.1% | | | 19 | | | | 14.6% | | 42 | | | 11.8% | | |  |  |  |  |
| 4 | | 25 | 11.0% | | | 7 | | | | 5.4% | | 32 | | | 9.0% | | |  |  |  |  |
| 5 | | 30 | 13.2% | | | 10 | | | | 7.7% | | 40 | | | 11.2% | | |  |  |  |  |
| 6 | | 17 | 7.5% | | | 17 | | | | 13.1% | | 34 | | | 9.5% | | |  |  |  |  |
| 7 | | 21 | 9.3% | | | 14 | | | | 10.8% | | 35 | | | 9.8% | | |  |  |  |  |
| 8 | | 20 | 8.8% | | | 9 | | | | 6.9% | | 29 | | | 8.1% | | |  |  |  |  |
| 9 | | 23 | 10.1% | | | 9 | | | | 6.9% | | 32 | | | 9.0% | | |  |  |  |  |
| 10 (least deprived) | | 14 | 6.2% | | | 11 | | | | 8.5% | | 25 | | | 7.0% | | |  |  |  |  |
| *missing* | | *14* |  | | | *42* | | | |  | | *56* | | |  | | |  |  |  |  |
| Total | | 241 |  | | | 172 | | | |  | | 413 | | |  | | |  |  |  |  |

**Table S2 – Detailed costing of delivering prenatal exome sequencing (pES)**

| **Process** | **Resource description** | **Unit cost (£)** | **Units** | **No. of units** | **Resource weighting** | **Total cost per case (£)** | **No. of cases** | **Total cost (£)** | **Source** |
| --- | --- | --- | --- | --- | --- | --- | --- | --- | --- |
| **Case identification and referral to GLH** | Multi-disciplinary team meeting | 337.88 | case | 1 | n/a | **338** | **413** | **139,543** | Taylor et al. 2019 (low cost £321)^2^ |
| **Eligibility review by GLH (rejected/non-proceeded)*** | Clinical scientist | 0.72 | minutes | 15 | n/a | **11** | **163** | **1,766** | Estimate (testing GLH) |
| **Consent** | FMU Consultant | 1.64 | minutes | 32 | 10.5% | 6 |  |  | EXPRESS survey data |
|  | Clinical Geneticist | 1.64 | minutes | 32 | 46.2% | 25 |  |  |  |
|  | Genomic Counsellor | 0.72 | minutes | 32 | 37.4% | 9 |  |  |  |
|  | Genomics Associate | 0.47 | minutes | 32 | 5.9% | 1 |  |  |  |
|  |  |  |  |  |  | **40** | **250** | **9,935** |  |
| **Sample collection, transport and DNA extraction** | Phlebotomy appointment | 4.70 | appointment | 2 | n/a | 9 |  |  | NHS reference costs 21/22^3^ |
|  | Transport | 5.00 | delivery | 2 | n/a | 10 |  |  | Estimate |
|  | Laboratory DNA extraction | 28.07 | DNA extraction | 2 | n/a | 56 |  |  | Testing GLH costing |
|  |  |  |  |  |  | **76** | **250** | **18,887** |  |
| **Prenatal Exome Sequencing (pES)** | Trio/duo exome sequence | 2,930.93 | trio(/duo) pES | 1 | n/a | **2,931** | **250** | **732,733** | Testing GLH costings |
| **Return of results & follow-up** | FMU Consultant | 1.64 | minutes | 113 | 14.9% | 28 |  |  | EXPRESS survey data |
|  | Clinical Geneticist | 1.64 | minutes | 113 | 51.1% | 95 |  |  |  |
|  | Genomic Counsellor | 0.72 | minutes | 113 | 27.6% | 23 |  |  |  |
|  | Obstetrician | 1.64 | minutes | 113 | 1.3% | 2 |  |  |  |
|  | FM Midwife | 0.72 | minutes | 113 | 5.1% | 4 |  |  |  |
|  |  |  |  |  |  | **151** | **241** | **36,498** |  |
| **Additional admin (across whole pathway)** | FMU Consultant | 1.64 | minutes | 46 | 10.5% | 8 |  |  | EXPRESS survey data |
|  | Clinical Geneticist | 1.64 | minutes | 46 | 46.2% | 35 |  |  |  |
|  | Genomic Counsellor | 0.72 | minutes | 46 | 37.4% | 12 |  |  |  |
|  | Genomics Associate | 0.47 | minutes | 46 | 5.9% | 1 |  |  |  |
|  |  |  |  |  |  | **57** | **413** | **23,366** |  |
| **Grand total** | | | | | | | | **962,727** |  |

* For accepted cases this cost is included in pES

**Table S3 – Estimated incremental annual cost to NHS**

| **Case type** | **No. of cases** | **Cost per case (£)** | **Total cost (£)** |
| --- | --- | --- | --- |
| Referred (case identification and referral, admin) | 760 | 394 | 299,782 |
| Proceeded (consent through to return of results) | 442 | 3,198 | 1,413,366 |
| Non-proceeded (GLH eligibility review only) | 301 | 11 | 3,260 |
| Non-proceeded (consent through to sequencing, then transferred to non-urgent pathway) | 17 | 3,046 | 51,786 |
|  |  |  | **1,768,193** |

1. Peter M, Mellis R, McInnes-Dean H, Daniel M, Walton H, Fisher J, et al. Delivery of a national prenatal exome sequencing service in England: a mixed methods study exploring healthcare professionals’ views and experiences. Front Genet. 2024 Jun 5;15.

2. Taylor J, Craft J, Blair E, Wordsworth S, Beeson D, Chandratre S, et al. Implementation of a genomic medicine multi-disciplinary team approach for rare disease in the clinical setting: a prospective exome sequencing case series. Genome Med. 2019 Dec 25;11(1):46.

3. NHS England. 2021/22 National Cost Collection Data Publication [Internet]. 2024 [cited 2024 Feb 23]. Available from: https://www.england.nhs.uk/publication/2021-22-national-cost-collection-data-publication/
